# Downregulated Expression of Solute Carrier Family 26 Member 6 in NRK-52E Cells Attenuates Oxalate-Induced Intracellular Oxidative Stress

**DOI:** 10.1155/2018/1724648

**Published:** 2018-10-10

**Authors:** Hongyang Jiang, Xintao Gao, Jianan Gong, Qian Yang, Ruzhu Lan, Tao Wang, Jihong Liu, Chunping Yin, Shaogang Wang, Zhuo Liu

**Affiliations:** ^1^Department of Urology, Tongji Hospital, Tongji Medical College, Huazhong University of Science and Technology, Wuhan, 430030 Hubei, China; ^2^Institute of Urology, Tongji Hospital, Tongji Medical College, Huazhong University of Science and Technology, Wuhan, 430030 Hubei, China; ^3^Department of Nephrology, Tongji Hospital, Tongji Medical College, Huazhong University of Science and Technology, Wuhan, 430030 Hubei, China; ^4^School of Pharmacy, Tongji Medical College, Huazhong University of Science and Technology, Wuhan, 430030 Hubei, China

## Abstract

Solute carrier family 26 member 6 (Slc26a6), which is mainly expressed in the intestines and kidneys, is a multifunctional anion transporter that is crucial in the transport of oxalate anions. This study is aimed at investigating the effect of Slc26a6 expression on oxalate-induced cell oxidation and crystal formation. Lentivirus transfection was used to upregulate or downregulate Slc26a6 expression in NRK cells. Cell viability and apoptosis, reactive oxygen species (ROS) and malondialdehyde (MDA) generation, and superoxide dismutase (SOD) activity were measured. Crystal adhesion and the cell ultrastructure were observed using light and transmission electron microscopy (TEM). Three groups of rats, normal control, lentivirus-vector, and lentivirus-small interfering RNA (lv-siRNA) groups, were used, and after lentivirus transfection, they were fed 1% ethylene glycol (EG) and 0.5% ammonium chloride (NH_4_Cl) for 2 weeks. Dihydroethidium (DHE), terminal deoxynucleotidyl transferase (TdT) deoxyuridine dUTP nick-end labeling (TUNEL), and von Kossa staining were performed, and nuclear factor *κ*B (NF*κ*B) and osteopontin (OPN) expression were measured. In the vitro study, compared to the control group, downregulated Slc26a6 NRK cells showed alleviation of the cell viability decrease, cell apoptosis rate, ROS generation, and SOD activity decrease after oxalate treatment. Crystal adhesion and vesicles were significantly less after oxalate exposure than in the untreated controls. Rats infected with lentivirus-siRNA exhibited attenuated SOD generation, cell apoptosis, and crystal formation in the kidneys. Increased phosphorylation of NF*κ*B and OPN was involved in the pathological process. In conclusion, the results of the present study indicate that reducing the expression of Slc26a6 in the kidney may be a potential strategy for preventing stone formation.

## 1. Introduction

Urolithiasis is a common disease caused by a combination of metabolic and genetic risk factors [1, 2] which is characterized by the development of stony concretions in the bladder or urinary tract. Calcium oxalate (CaOx) stones are the most prevalent type of kidney stones, accounting for 70–80% of incidences [3]. Hyperoxaluria is a major risk factor for CaOx urolithiasis and is the result of both genetic and environmental factors. Enhanced absorption of oxalate by the intestine, internal production of oxalate by the liver, and excretion of oxalate by the kidney all contribute to hyperoxaluria [4]. High oxalate accumulation in the renal tubules induces CaOx crystal formation and renal cell injury, which promote the progression of CaOx urolithiasis.

Numerous studies have confirmed an association between CaOx crystal deposition in the kidneys and renal epithelial injury since Randall emphasized the importance of renal damage in stone formation [5, 6]. Many studies have indicated that oxidative stress and inflammation are important pathologies for kidney stone formation [7, 8]. Clinical studies have also reported that the kidneys of patients with CaOx stones were under oxidative stress [9]. Renal tubular epithelial cell overproduction of reactive oxygen species (ROS) is attributable to oxidative stress under conditions of high oxalate stimulation, which triggers epithelial cell injury, inflammation, and ultimately, cell apoptosis [10, 11]. ROS-induced cell injury is an initial mechanism of crystal nucleation, attachment, and retention [7, 12, 13].

Nuclear factor-*κ*B (NF*κ*B) is a nuclear transcription factor involved in inflammation and immune responses. NF*κ*B usually exists in the cytoplasm in the form of dimers. Inhibitor of NF*κ*B (I*κ*B) binds to the NF*κ*B dimer to inhibit NF*κ*B phosphorylation and activation [14]. Following stimulation by cytokines or ROS, I*κ*B*α* is phosphorylated and depolymerized with the NF*κ*B dimer. The released NF*κ*B dimers are activated by phosphorylation and can then be transported to the nucleus to regulate gene expression [15]. Osteopontin (OPN) plays an important role in the development of urolithiasis. Oxalate induces OPN expression by activating NF*κ*B in tubular cells [16]. OPN also induces NF*κ*B activation through the phosphorylation and degradation of I*κ*B*α* by inducing IK kinase activity [14].

Solute carrier family 26 member 6 (Slc26a6) is an important protein that mediates oxalate transport, and it is expressed mainly in the apical membrane of the intestine and kidneys. In the intestine, Slc26a6 on the apical membrane of intestinal epithelial cells can transport Ox^2−^ from the blood to the intestinal lumen (oxalate secretion) through Cl/Ox^2−^ exchange [17]. In the kidneys, Slc26a6 is located in the proximal tubular epithelial cell side of the renal tubular cell and can transfer oxalate from the blood to the urine through the Cl^−^/Ox^2−^ exchange (oxalate secretion) [18].

Ox^2−^ also can be transferred from the urine to blood via SO_4_^2−^/Ox^2−^ exchange (oxalate reabsorption). Many studies have shown that the level of Slc26a6 expression is highly correlated to oxalate homeostasis [19]. Based on these theories and studies, we hypothesized that the expression of Slc26a6 in NRK-52E cells could affect oxalate absorption and activate the NF*κ*B/OPN pathway by regulating the degree of oxidative stress. In this study, we attempted to upregulate and downregulate Slc26a6 expression in NRK cells to explore whether it plays a key role in ROS production in NRK cells stimulated with high oxalate.

## 2. Materials and Methods

### 2.1. Cell Culture

The normal rat proximal tubular epithelial cell line (NRK-52E) was obtained from the Type Culture Collection of the Chinese Academy of Sciences (Shanghai, China). Cells were maintained in Dulbecco's modified Eagle's medium (DMEM) (HyClone, CT, USA) supplemented with 10% fetal bovine serum (Gibco, Grand Island, NY, USA) at 37°C under a humidified 5% CO_2_ atmosphere.

### 2.2. Construction of Lentivirus with Slc26a6 or siRNA-Slc26a6

The Slc26a6 sequence (gene ID: 301010) was chosen according to the National Center for Biotechnology Information (NCBI) GenBank. To knock down Slc26a6 expression, lentiviral vector piLenti-siRNA-red fluorescent protein- (RFP-) based short hairpin RNA (shRNA) against Slc26a6 was constructed, and the targeting sequence of shSlc26a6 was 5′-GGGAACTACTCAAGCTAAT-3′ [17]. The sequences with red fluorescent protein (RFP) were synthesized and inserted into the vector pWSLV and pilentivirus as pWSLV-05-Slc26a6 and piLenti-siRNA (Slc26a6), respectively. The plasmid containing the coding sequence of Slc26a6 was purchased from Genecopoeia™, and 293T cells were seeded in 10 cm dishes until they reached 80–90% confluence. The transfection complex was directly added to each dish, and the cells were incubated in a CO_2_ incubator at 37°C overnight. A lentivirus particle-containing culture medium was harvested 48 h post-transfection. The final titers of the lentiviral vector were determined to contain 10^8^ TU/mL.

### 2.3. Stable Transfection of NRK-52E Cells

pWSLV-05-Slc26a6, piLenti-siRNA (Slc26a6), and pLenti-vector were directly transfected into NRK-52E cells, and the medium was removed after 24 h. For stable transfection, the cells were cultured in DMEM medium containing 2.0 *μ*g/mL puromycin (Beyotime Biotechnology, Shanghai, China) and the selective medium was changed after 48 h. The NRK cells in our experiment were divided into normal (NRK-52E), lv-Slc26a6 transgenic (NRK-Slc26a6), siRNA-Slc26a6 transgenic (NRK-siRNA), and vector transgenic (NRK-vector) NRK cells.

### 2.4. Western Blotting

Cells were lysed in NP-40 with 1.0 mM protease inhibitor phenylmethanesulfonyl fluoride (PMSF) (Beyotime Biotechnology, Shanghai, China) and 1.0 mM phosphoproteinase inhibitors (Servicebio, Hubei, China). The protein concentration was measured using the bicinchoninic acid (BCA) protein assay kit (Beyotime Biotechnology, Shanghai, China). After diluting the protein sample with 5x loading buffer and boiling for 10 min, it was loaded equally on a sodium dodecyl sulfate- (SDS-) 10% polyacrylamide gel and electrophoresed, and then the proteins were transferred onto polyvinylidene fluoride (PVDF) membranes. The PVDF membranes were blocked with 5% bovine serum albumin (BSA) for 2 h and probed with primary antibodies against Slc26a6 (1 : 200, sc-26728, Santa Cruz Biotechnology, CA, USA), NADPH oxidase 2 (Nox2, 1 : 200, 19013-1-AP, Proteintech, Hubei, China), Nox4 (1 : 200, 14347-1-AP, Proteintech, Hubei, China), NF*κ*B p65 (1 : 500, GB11142, Servicebio, Hubei, China), NF*κ*B-p65 (phosphorylated [p]-Ser536, 1 : 500, 11014, Signalway Antibody Co., Ltd, MD, USA), I*κ*B*α* (1 : 500, GB13212-1, Servicebio, Hubei, China), I*κ*B*α* (p-Ser32/36, 1 : 500, 11152, Signalway Antibody Co., Ltd, MD, USA), and OPN (1 : 200, 22952-1-AP, Proteintech, Hubei, China) at 4°C overnight. After three washes with Tris-buffered saline plus Tween (TBST), the PVDF membranes were incubated with HRP-conjugated anti-goat and anti-rabbit antibodies (1 : 5000, Boster Biological Technology Co., Ltd, China) for 2 h. Finally, the membranes were washed with TBST three times, and the blots were visualized with enhanced chemiluminescence (ECL) reagent using Bio-Rad Clarity Western ECL substrate (Bio-Rad Laboratories, CA, USA). Anti-*β*-actin was used to normalize the protein expression, and each Western blot was repeated in triplicate.

### 2.5. Immunofluorescence (IF)

Cells were cultured until they reached 80% confluence, and then they were fixed with 4% paraformaldehyde for 15 min, followed by permeabilization with 0.5% Triton X-100 for 20 min and blocking with BSA for 30 min. After washing with phosphate-buffered saline (PBS), the cells were incubated with an anti-Slc26a6 antibody (1 : 50) overnight at 4°C and then probed with anti-goat IgG FITC for 1 h at 25°C. The cells were washed in PBS plus Tween (PBST), treated with 4′,6-diamidino-2-phenylindole (DAPI) for 5 min, and then the fluorescent signals of both assays were observed using a BX53 microscope (Olympus, Tokyo, Japan). The fluorescence intensities were determined using the ImageJ software.

### 2.6. Real-Time qPCR

Using the TRIzol method, total RNA was extracted from cells, and then cDNA was synthesized using the cDNA Master Mix for quantitative polymerase chain reaction (qPCR, Takara Bio Inc., Japan) following the manufacturer's instructions. PCR was performed in a reaction volume of 20 *μ*L containing SYBR Green using a CFX Connect Real-Time PCR System (Bio-Rad, CA, USA). The level of *β*-actin mRNA expression was measured as an endogenous reference and used for normalization. The results were calculated using the 2^−∆∆Ct^ method to analyze differences in mRNA expression levels between samples. The primers for Slc26a6 used in this experiment were as follows: forward, CTTCGGTTTTGTGGTCACCT and reverse, TTTAACCAGCACCAACACCA.

### 2.7. Lactate Dehydrogenase (LDH) Assay

Total intracellular lactate dehydrogenase (LDH) was measured to detect the cell injury using an LDH measuring kit (C0017, Beyotime Biotechnology, Shanghai, China). Cells were seeded in 96-well plates at a concentration of 5 × 10^3^ cells/well, and the medium was replaced with DMEM without FBS, followed by treatment with or without 700 *μ*M oxalate [20] for 12 h. Then the supernatants were collected and measured according to the instructions to determine the LDH release activity, which was presented as the fold increase over the control group levels.

### 2.8. Cell Viability Detection Using CCK-8

Different cells were seeded at a concentration of 5 × 10^3^ cells/well in 96-well plates and incubated overnight. The cells were treated with or without oxalate (700 *μ*M) for 72 h [20]. During the treatment, the cell viability change was measured using a CCK-8 assay kit according to the instructions.

### 2.9. Lipid Peroxidation (Malondialdehyde, MDA) Measurement

Lipid peroxidation was measured as the malondialdehyde (MDA) content, which was determined using the kit according to the instructions (S0131, Beyotime Biotechnology, Shanghai, China). The MDA increment rates were expressed as percentages (%).

### 2.10. Superoxide Dismutase (SOD) Activity Measurement

Superoxide dismutase activity (SOD) was measured by kit (S0101, Beyotime Biotechnology, Shanghai, China), and the protocol was performed according to the manufacturer's instructions. The change in SOD activity from with to without oxalate treatment was expressed as a percentage (%).

### 2.11. Annexin V/PI Staining and Flow Cytometric Analysis

The early and late apoptotic stages of four kinds of cells were identified and quantified using an Annexin V-FITC/PI detection kit (40303, Yeasen, Shanghai, China). After exposure to oxalate (700 *μ*M) for 24 h, the early and late apoptotic cells were quantified [20]. The protocol was performed according to the manufacturer's instructions. The apoptotic cells increment rate was expressed as %.

### 2.12. Preparation of Ultrathin Sections for TEM

After 700 *μ*M oxalate treatment for 24 h, cells were fixed in 5% glutaraldehyde at 4°C [21]. The cells were post-fixed with 1% osmium tetroxide for 1 h and embedded in epoxy resin. Ultrathin sections were subsequently stained with aqueous uranyl acetate/lead citrate and observed using an FEI transmission electron microscope (TEM) at 200 kV at the Center for Instrumental Analysis and Metrology, Institute of Virology, Chinese Academy of Science.

### 2.13. Detection of Total Intracellular ROS

Intracellular ROS production was detected using an ROS assay kit (50101, Yeasen, Shanghai, China). Briefly, cells were seeded in six-well plates and treated with or without 700 *μ*M of oxalate. After 3 h, the cells were treated with 2,7-dichlorodihydrofluorescein diacetate (DCFH-DA, 10 *μ*M) for 30 min. Then, the cells were collected and suspended in 200 *μ*L PBS. Intracellular ROS levels were measured using flow cytometry (BD Biosciences, NY, USA), and all determinations were performed in triplicate. The ROS generation increment rate was expressed as a percentage (%).

### 2.14. Animal Experiments

Nine male Sprague-Dawley rats (275–300 g) were obtained from the Animal Center of Tongji Medical College, Huazhong University of Science and Technology. They were reacclimatized to a 12 h light/dark cycle at 23°C for 1 week prior to the start of the experiments in a specific pathogen-free animal house with a relative humidity of 45%–55%. They were allowed free access to a standard laboratory chow diet and were randomly divided into three groups of three rats each: normal control (NC), siRNA, and vector groups. The experimental protocol was conducted in accordance with the institutional ethical committee of Tongji Hospital, Tongji Medical College, Huazhong University of Science and Technology, according to the “Guidelines for Experimental Animal Ethical Committee of Huazhong University of Science and Technology.”

### 2.15. Lentivirus Transfection

The rats were placed in the prone position and anesthetized with sodium pentobarbital (40 mg/kg). Lentivirus subcapsular renal transfection was accomplished according to a previously published protocol [22], and every effort was made to minimize animal suffering. The NC, siRNA, and vector groups were injected with physiological saline, lentivirus-siRNA, and lentivirus-vector, respectively.

### 2.16. Detection of Crystal Formation

Ethylene glycol (EG) and ammonium chloride (NH_4_Cl) were dissolved in water to 1% and 0.5% concentrations, respectively. After a 2-week treatment, the rats were euthanized under anesthesia, the kidneys were removed, and von Kossa staining was used to identify crystal formation.

### 2.17. Detection of ROS

The rat kidney tissues were quickly frozen, cut to a thickness of 8 *μ*m at an optimized cutting temperature, and mounted on glass slides [23]. Superoxide generation was measured by staining the tissue with dihydroethidium (DHE) for 30 min protected from light. Fluorescence images were captured using an Olympus BX53 fluorescence microscope (Olympus, Tokyo, Japan).

### 2.18. Terminal Deoxynucleotidyl Transferase dUTP Nick-End Labeling (TUNEL) Assay

The terminal deoxynucleotidyl transferase dUTP nick-end labeling (TUNEL) assay was conducted using a TUNEL detection kit according to the manufacturer's instructions (11684817910, Roche, Basel, Switzerland). Briefly, sections were incubated with 0.1% Triton X-100 and 0.1% sodium citrate for 8 min at room temperature and then washed with TBS. This was followed by incubation with a mixture of 3% BSA and 20% calf serum for 30 min at room temperature. After removing the serum, TUNEL reaction liquid was added to the sections, which were incubated for 60 min at 37°C, and then washed with TBS. The nuclei were counterstained with DAPI (Servicebio, Hubei, China). Images were captured using an Olympus BX53 fluorescence microscope (Olympus, Tokyo, Japan).

### 2.19. Statistical Analysis

The results were analyzed using GraphPad Prism 6.0 (GraphPad Software, San Diego, CA, USA) and are presented as means ± standard deviation (SD). An unpaired *t*-test was performed to analyze the differences between any two groups, and differences were considered significant at *P* < 0.05.

## 3. Results

### 3.1. Transgenic Cell Confirmation

**Figure 1:**
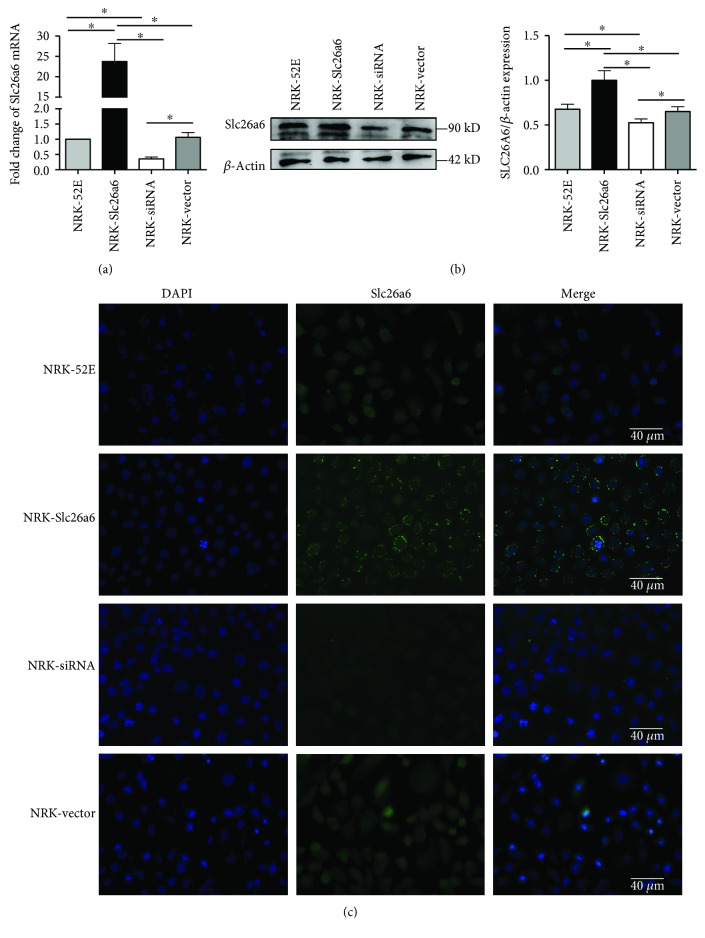
Transfection of lentivirus regulated Slc26a6 expression of NRK-52E. (a) Slc26a6 expression level with *β*-actin as the loading control in kidney tissues of all groups using real-time PCR. The values are presented as mean ± SD and as fold change relative to control. (b) Representative Western blot results of Slc26a6 of rats in the NRK-52E, NRK-Slc26a6, NRK-siRNA, and NRK-vector groups after lentivirus infection. Expression levels of Slc26a6 with *β*-actin as the loading control in all the four groups are presented as a bar graph. Data are means ± SD. ^∗^*P* < 0.05. (c) Representative images of immunofluorescence (IF) assays to detect Slc26a6 in all four groups (scale bar, 40 *μ*m).

According to the results of the quantitative polymerase chain reaction (qPCR), Western blotting, and immunofluorescence (IF), lentivirus-small interfering RNA (lv-siRNA) reduced Slc26a6 expression successfully in NRK cells while lv-Slc26a6 increased the expression (Figure 1).

### 3.2. Effects of Oxalate and Slc26a6 Expression on NRK-52E Cell Viability

**Figure 2:**
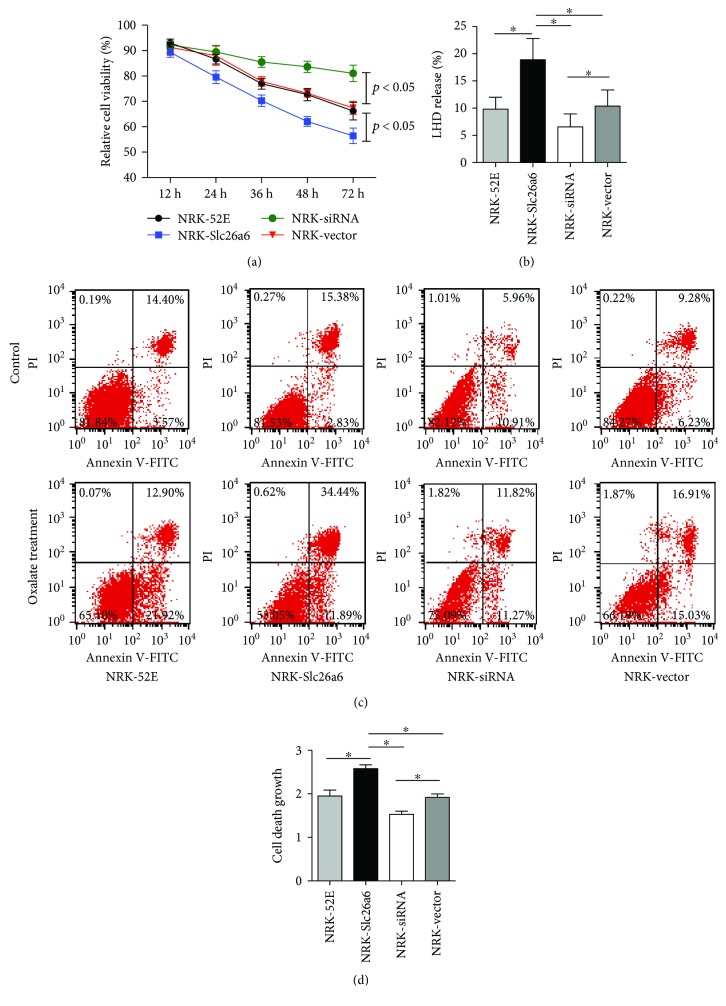
High Slc26a6 expressed in NRK cells promotes oxalate-induced cell injury. Different cell types were treated with 700 *μ*M oxalate. (a) Cell viability was measured using CCK-8 assay, and growth of NRK-Slc26a6 cells decreased more significantly than that of NRK-52E while NRK-siR attenuated this reduction. Data are means ± SD (*n* = 6). ^∗^*P* < 0.05. (b) LDH release was measured to evaluate the cell toxicity of oxalate, and the NRK-Slc26a6 group showed more toxicity than controls did. Data are means ± SD (*n* = 6). ^∗^*P* < 0.05. (c) Representative images of apoptotic cells at 48 h, detected using flow cytometry before and after oxalate treatment. PI: propidium iodide. (d) Cell death changes are expressed as means ± SD in the column graph (*n* = 3). ^∗^*P* < 0.05.

After exposure to oxalate, the cells were analyzed using the cell counting kit-8 (CCK-8) assay, which indicated that the viability of NRK-Slc26a6 cells was decreased more than that of other groups, while a lower Slc26a6 expression ameliorated the reduction of cell viability (Figure 2(a)).

### 3.3. Higher Slc26a6 Expression Increased Oxalate-Mediated Cell Injury

To further analyze the effect of lower Slc26a6 levels on oxalate-induced cell injury, the LDH release activity was detected. Exposure of the cells to oxalate (700 *μ*M) for 12 h caused the release of LDH in the group with higher Slc26a6 expression (Figure 2(b)). In contrast, the siRNA-Slc26a6 transgenic NRK cell (NRK-siRNA) group showed a higher decrease in the oxalate-induced LDH leakage than that of the control groups (normal NRK cells [NRK-52E] and vector transgenic NRK cells [NRK-vector]).

### 3.4. Higher Slc26a6 Expression Increased Oxalate-Mediated Cell Apoptosis

The fluorescein isothiocyanate (FITC)/propidium iodide (PI) apoptosis assay was performed to assess the mode of cell death caused by oxalate toxicity. FITC/PI staining and flow cytometry analysis revealed that 81.00 ± 1.90, 82.32 ± 0.80, 83.00 ± 0.92, and 82.85 ± 1.37% of the untreated NRK-52E, lv-Slc26a6 transgenic NRK (NRK-Slc26a6), NRK-siRNA, and NRK-vector cells, respectively, were viable. After exposure to oxalate for 24 h, viable cells were significantly decreased. The number of viable cell was reduced to 63.44 ± 2.18% (NRK-52E), 53.70 ± 2.02% (NRK-Slc26a6), 73.21 ± 1.75% (NRK-siRNA), and 66.83 ± 1.81% (NRK-vector; Figure 2(c)). The rate of increase in apoptotic cells was expressed as a graph in Figure 2(d).

### 3.5. Higher Slc26a6 Expression Increased Crystal Adhesion to Cells

**Figure 3:**
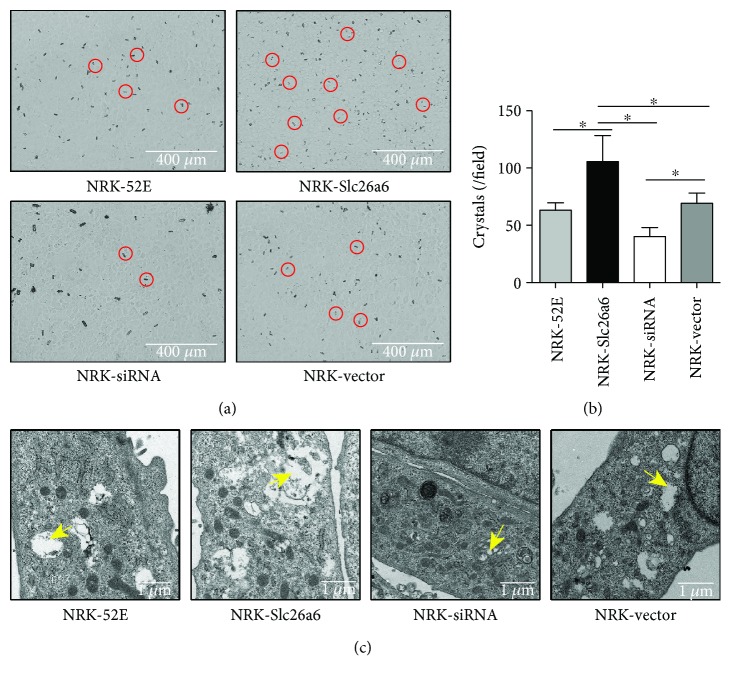
Less Slc26a6 expression induced less oxalate crystal adhesion to cells and less intracellular vesicles. (a) Crystals were microscopically measured after a 24 h exposure. Red circle, one crystal. Magnification, ×100. (b) COM levels of cells. Data are means ± SD (*n* = 6). ^∗^*P* < 0.05. (c) After a 24 h oxalate treatment, ultrastructural observations using TEM. The micrographs showed that smaller intracellular vesicles were produced in NRK-siRNA. Yellow arrow indicates vesicles (TEM, ×5000).

After exposure to oxalate (700 *μ*M) for 24 h, a light microscope was used to observe the crystal formation. The results showed that the higher Slc26a6 (NRK-Slc26a6) group accumulated the most calcium oxalate monohydrate (COM) cell adhesion, whereas NRK-siRNA had the least (Figure 3(a)).

### 3.6. Oxalate Induced More Intracellular Vesicles in the Higher Slc26a6 Group in Transmission Electron Microscopy (TEM) Analysis

After treatment with 700 *μ*M oxalate for 24 h, fixed cells were observed using transmission electron microscopy (TEM), which showed that bigger intracellular vesicles were produced in the higher Slc26a6 group than in the control group. Compared to the control groups, smaller intracellular vesicles and more normal-shaped cells were observed in the lv-siRNA group (Figure 3(b)). No apparent changes were observed in the NRK-52E and NRK-vector groups.

### 3.7. Higher Slc26a6 Expression Induced Higher ROS Production

**Figure 4:**
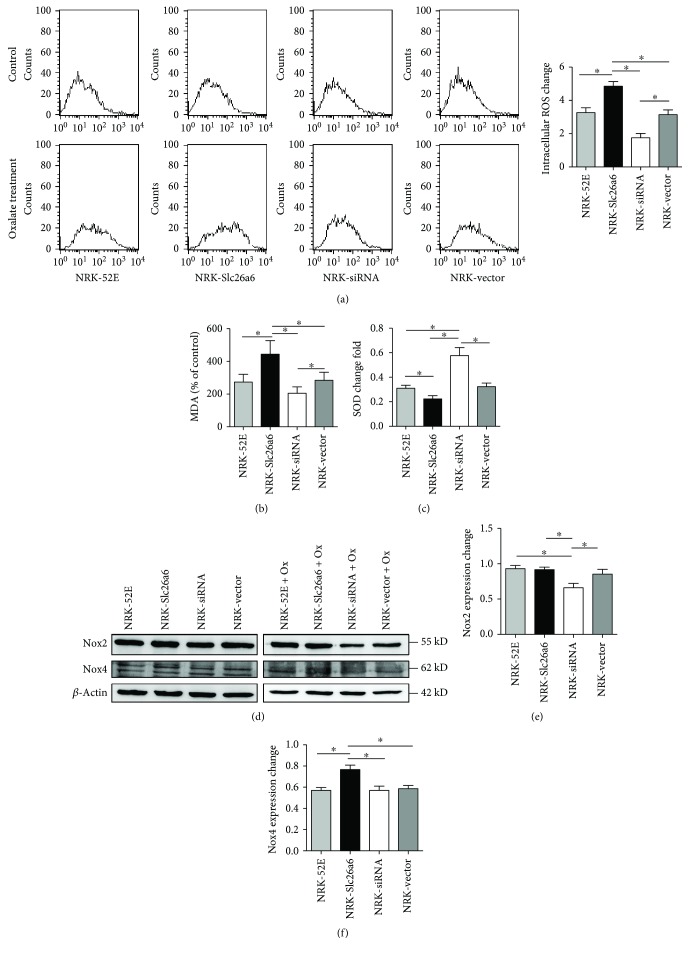
Higher Slc26a6 expression induced more intracellular ROS generation by oxalate. (a) Four cell types were stimulated with oxalate (700 *μ*M) for 3 h, and intracellular ROS levels were determined by fluorescence intensity of DCFH analyzed using flow cytometry. Changes before and after oxalate treatment are shown on the right graph as means ± SD (*n* = 3). ^∗^*P* < 0.05. (b) Lipid peroxidation was assessed by detecting the MDA level in supernatants of NRK cell lysates. NRK-Slc26a6 cells showed an obvious increase in MDA generation compared with control groups after oxalate treatment (*n* = 6). (c) Activity change of superoxide dismutase (SOD) expressed as means ± SD. In the NRK-Slc26a6 group, SOD activity was markedly decreased compared to that in control groups. (d, e, f) Expressions of NADPH oxidase 2 (Nox2) and Nox4 before and after oxalate treatment in all groups using Western blot analysis. After oxalate treatment (700 *μ*M) for 24 h, the expression of Nox4 increased in the NRK-Slc26a6 group and that of Nox2 was decreased in the NRK-siRNA group. Data are means ± SD (*n* = 3), ^∗^*P* < 0.05.

The DCFH-DA fluorescence was measured to determine the total intracellular ROS levels. NRK-Slc26a6 significantly increased the ROS generation compared with NRK-52EE and NRK-vector after oxalate exposure (Figure 4(a)).

### 3.8. Higher Slc26a6 Expression Enhanced Lipid Peroxidation Injury in Cells Exposed to Oxalate

MDA levels and SOD activity were examined as markers of lipid peroxidation injury and oxidative stress. The MDA assay indicated that NRK-Slc26a6 enhanced the lipid peroxidation injury in cells exposed to oxalate (Figure 4(b)). In the NRK-Slc26a6 group, the SOD activity was markedly decreased compared to that in the control groups (Figure 4(c)). Protein expression of NADPH oxidase (Nox) subunits changed in the different groups.

Before oxalate treatment, the Western blot showed that the four cell types expressed similar Nox2 and Nox4 levels. After exposure to 700 *μ*M oxalate for 24 h, the four cell types were lysed to measure Nox2 and Nox4. Compared to the NRK-52EE cells and NRK-vector, NRK-siRNA expressed less Nox2 and NRK-Slc26a6 expressed more Nox4 protein than the controls did (Figure 4(d)).

### 3.9. Less Slc26a6 Expression Decreased p-p65 and p-I*κ*B*α* Expression in Cells Exposed to Oxalate

**Figure 5:**
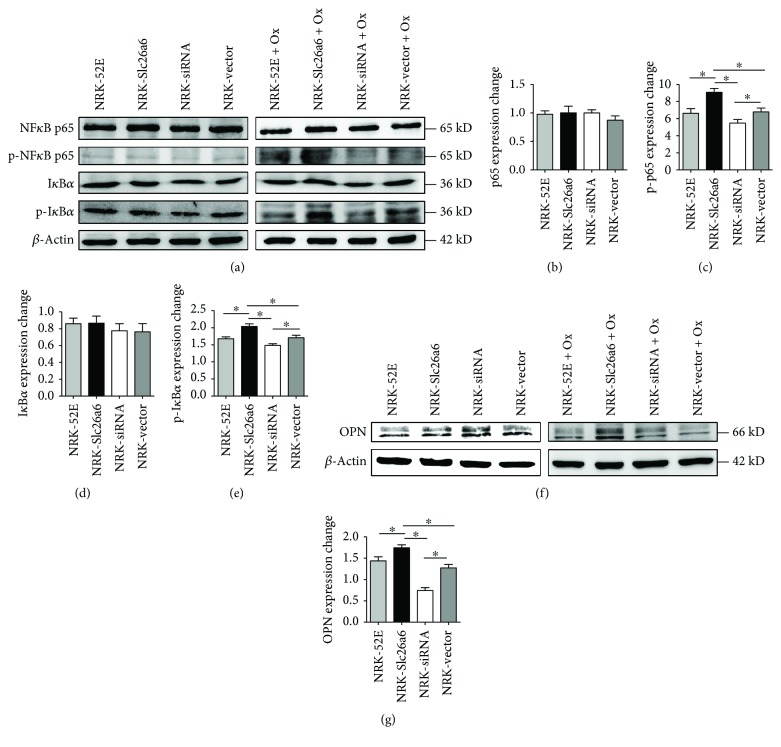
Expression of NF*κ*B, I*κ*B*α*, and OPN before and after oxalate exposure. (a) Expressions of p65, p-p65, I*κ*B, and p-I*κ*B*α* in four cell types using Western blot analysis. (b–e) Change in protein expression before and after oxalate treatment. Data are means ± SD(*n* = 3), ^∗^*P* < 0.05. (f, g) Representative Western blots of OPN in four cell types using Western blot analysis. Data are means ± SD (*n* = 3), ^∗^*P* < 0.05.

The expressions of p65, p-p65, I*κ*B*α*, and p-I*κ*B*α* in cells were measured using a Western blot assay. The results indicated that the four cell types expressed similar p65, p-p65, I*κ*B*α*, and p-I*κ*B*α* protein levels before oxalate treatment, but the NRK-Slc26a6 group expressed more p-p65 and p-I*κ*B*α* and NRK-siRNA expressed less p-p65 and p-I*κ*B*α* than the control groups did (Figure 5(a)). The changes in protein expression levels between values before and after oxalate exposure are shown as plot graphs. p-p65 and p-I*κ*B*α* increased significantly in the NRK-Slc26a6 group and decreased in NRK-siRNA (Figures 5(b)–5(e)).

### 3.10. Less Slc26a6 Expression Decreased OPN Expression in Cells Exposed to Oxalate

A comparison of the OPN expression of cells before and after oxalate treatment showed that it was more significantly increased in N-26 cells than it was in control cells. However, NRK-siRNA ameliorated the increase compared to that of the control cells (Figures 5(f) and 5(g)).

### 3.11. lv-siRNA Subcapsular Transfection Reduced Expression of Slc26a6 in Kidney Tissue rather than Duodenum

**Figure 6:**
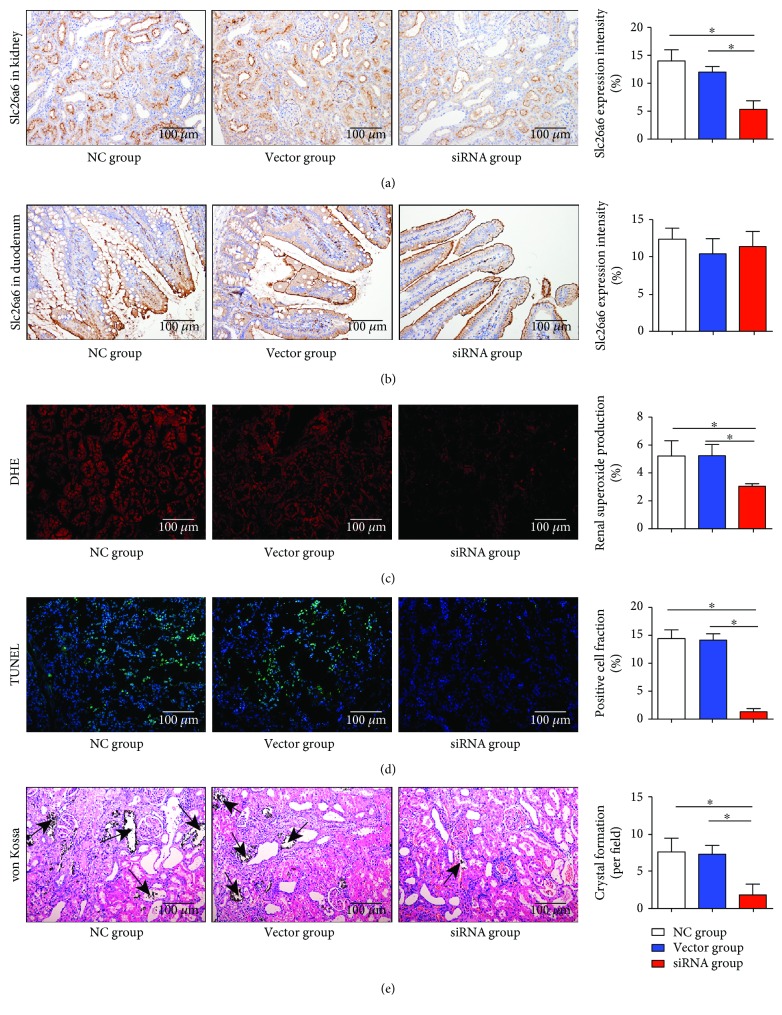
Less Slc26a6 expression in kidney protects rats from urolithiasis. After renal capsular transfection for 2 weeks, rats were treated with 1% EG and 0.5% NH_4_Cl. (a, b) IHC assay indicates that lentivirus-siRNA reduced the Slc26a6 expression in the kidney but not in the duodenum (*n* = 3). Magnification, ×200, ^∗^*P* < 0.05. (c) DHE staining was used to detect SOD generation, and the siRNA group showed higher decreases in SOD production than other groups did. Magnification, ×200, ^∗^*P* < 0.05. (d) Representative micrographs showing TUNEL staining images of different groups (*n* = 3). Magnification, ×200, ^∗^*P* < 0.05. (e) von Kossa staining to detect crystal formation and the siRNA group formed least crystals in the kidney. Arrows indicate crystals (*n* = 3). Magnification, ×200, ^∗^*P* < 0.05.

After lv-siRNA renal subcapsular injection, the Slc26a6 expression levels of the kidney and duodenum were analyzed using IHC. The result indicated that the lv-siRNA group expressed less Slc26a6 in the kidney than the other groups did, while levels in duodenum were not altered significantly (Figures 6(a) and 6(b)).

### 3.12. Lower Slc26a6 Expression in Rat Kidneys Induced Less SOD Generation and Cell Apoptosis

ROS production was detected in the three groups using DHE fluorescence. As shown in Figure 6(c), lower Slc26a6 expression in the rat kidneys produced less SOD than higher expression did. Furthermore, to assess cell injury and apoptosis, a TUNEL assay was performed, and the result (Figure 6(d)) indicated that the lower Slc26a6 expression in rat kidneys induced less cell apoptosis.

### 3.13. Lower Slc26a6 Expression in Rat Kidneys Induced Less Crystal Formation

To assess whether crystal formation was reduced after lv-siRNA transfection, von Kossa staining was performed, and the results indicate that the lv-siRNA group significantly attenuated the crystal formation after treatment with 1.0% EG and 0.5% NH_4_Cl treatment (Figure 6(e)).

### 3.14. Lower Slc26a6 Expression in Rat Kidneys Induced Less p-p65, p-I*κ*B*α*, and OPN Expression

**Figure 7:**
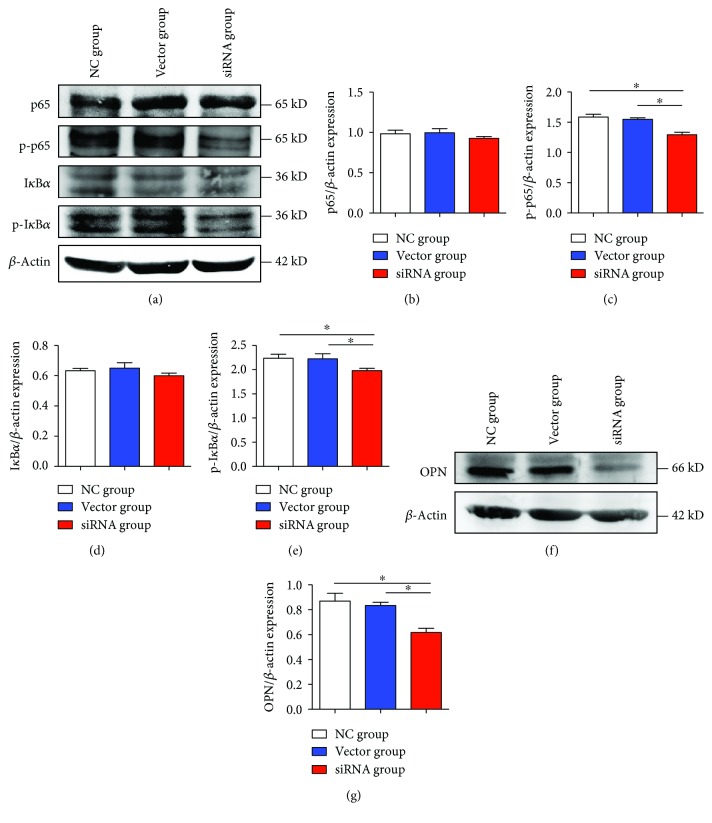
Expression of NF*κ*B, I*κ*B*α*, and OPN in rats. After renal capsular transfection for 2 weeks, rats were treated with 1% EG and 0.5% NH_4_Cl. (a–g) Representative Western blot of p65, p-p65, I*κ*B, p-I*κ*B*α*; OPN expression levels in three groups were detected using Western blotting, and the siRNA group expressed the lowest levels of p-p65, p-I*κ*B*α*, and OPN. Bar represents means ± SD of three independent experiments, ^∗^*P* < 0.05.

The Western blot analysis indicates that p-p65, p-I*κ*B*α*, and OPN were greatly decreased in the lv-siRNA-infected rats compared with levels in the control and lv-vector-infected rats, whereas p65 and I*κ*B*α* showed no significant difference among the three groups (Figure 7).

## 4. Discussion

The urinary oxalate concentration is closely related to the condition of the kidney, which is responsible for 90% of the oxalate excretion [19]. As a key oxalate transporter, Slc26a6 is closely related to oxalate homeostasis in vivo [24]. Studies have shown that mice without Slc26a6 expression exhibited significantly elevated serum and urinary oxalate levels [25]. The expression of Slc26a6 is somewhat correlated to stone formation [18, 26]. Slc26a6 expression in the kidney may account for the oxalate homeostasis and stone formation. The expression of Slc26a6 in NRK cells was upregulated and downregulated using lentivirus transfection in our study, and then the cells were stimulated with oxalate followed by measurement of cell viability and ROS production.

Both oxalate and COM crystals have abrasive effects on the renal epithelial cells through free radicals [13]. Oxalate can be excreted in urine through renal epithelial cells, and oxalate in urine is also reabsorbed by renal epithelial cells [27]. The oxalate transporter, Slc26a6, mediates the transport of oxalate into and out of the renal epithelial cell. The level of Slc26a6 expression could affect the oxalate absorption of cells. In our previous study, oxalate at a concentration of 700 *μ*M decreased NRK-52E cell viability and increased intracellular ROS production significantly [20]. In the present study, 700 *μ*M oxalate was used to detect the different cell characteristics, and the results indicate that the highest Slc26a6 group (NRK-Slc26a6) accumulated more crystals than the control group did. CaOx crystals injure cells by damaging cell membranes and producing lipid mediators and excessive ROS, which lead to an imbalance between oxidants and antioxidants, with malfunctioning of the mitochondria [28, 29]. The TEM analysis showed that NRK-Slc26a6 cells produced bigger intracellular vesicles than other cells did, which indicates more injury was induced in the NRK-Slc26a6 cells [30]. A previous in vitro study reported that cell membrane vesicles are implicated in crystallization [31]. To further verify the effect on cell viability, a CCK8 assay was performed, and NRK-Slc26a6 cells showed lower cell viability than the control cells did and NRK-siRNA ameliorated the reduced cell viability induced by oxalate treatment, which was consistent with the cell apoptosis measurement. The results of this study indicate that NRK cells with higher Slc26a6 expression (NRK-Slc26a6) exhibited higher levels of injury from oxalate exposure, while lower Slc26a6 expression caused less injury than that of the control.

ROS, which have been shown to play a principal role in many physiological and pathophysiological processes, can be produced by Nox [32]. Nox4 and Nox2 are the two predominant isoforms in the kidneys [20, 33, 34]. In contrast to Nox2, Nox4 produces hydrogen peroxide rather than superoxide [35]. Oxalate exposure regulated mRNA expression of Nox subunits in a renal epithelial-derived cell line [36]. Zhang et al. [20] demonstrated that MitoTEMPO, a mitochondria-targeted antioxidant, increased the protein expression of Nox4 compared with the control group exposed to oxalate [20]. Consistent with this conclusion, we observed that the protein expression of Nox4 was increased in NRK-Slc26a6 and Nox2 was decreased in NRK-siRNA after exposure to oxalate. Furthermore, ROS and MDA levels were increased by oxalate treatment in NRK-Slc26a6.

OPN is a phosphoprotein considered to be a key macromolecular modulator in the development of urolithiasis [37]. OPN expression is upregulated in the cortical tubules of the kidney after renal epithelium cell injury after stimulation with high concentrations of oxalate or COM adhesion [16, 38–40]. OPN plays a crucial role in the adhesion process of CaOx crystals to the renal tubular cells in stone formation [41]. Tozawa et al. reported that OPN could be upregulated by activation of the NF*κ*B signaling pathway [16].

NF*κ*B is a set of nuclear transcription factor proteins that usually exist in the cytoplasm in the form of dimers [15]. The most common forms of NF*κ*B are p65-p65 and p65-p50. I*κ*B binds to the NF*κ*B dimer to inhibit NF*κ*B phosphorylation activation. ROS lead to I*κ*B*α* phosphorylation and depolymerization with the NF*κ*B dimer, and the released NF*κ*B dimers are activated by phosphorylation [15]. The NF*κ*B signaling pathway also mediates OPN pathway activation, which is responsible for crystal adhesion [42]. Consistently, in our study, OPN, p-p65, and p-I*κ*B expression was induced in NRK-Slc26a6 after oxalate exposure, which indicates that the NRK-Slc26a6 group may have absorbed more oxalate or COM into cells than other groups did, and the NRK-siRNA cells expressed lower OPN protein and p-p65 and p-I*κ*B levels after oxalate treatment.

**Figure 8:**
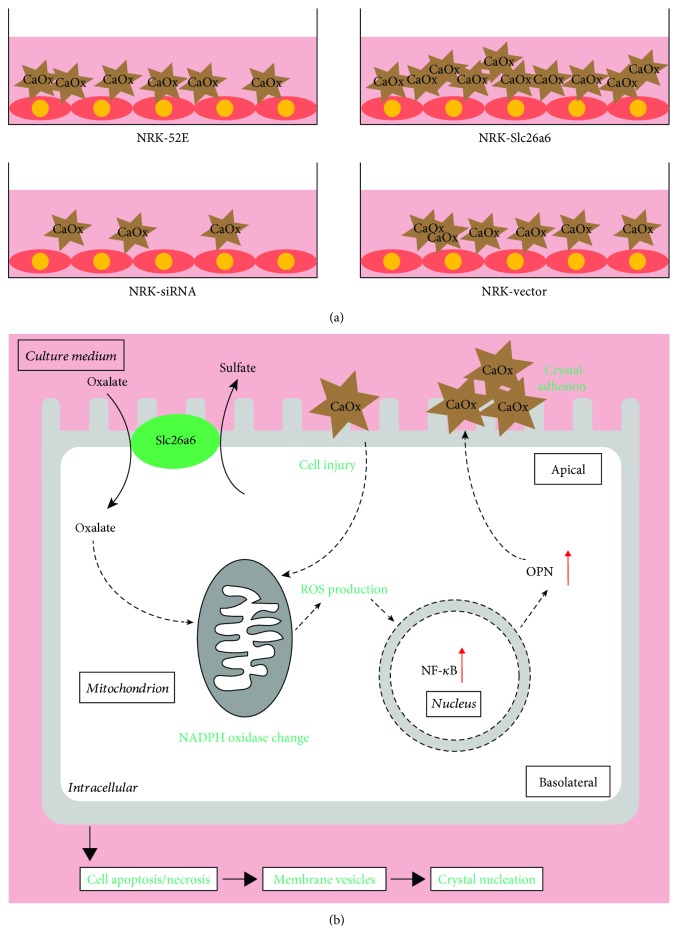
Proposed mechanisms of high Slc26a6-induced injury of renal tubular epithelium cells. (a) Slc26a6 expressed on the membrane of NRK cells attracts more calcium oxalate monohydrate (COM) adhesion to cells. (b) In renal tubular epithelium cells, high expression of Slc26a6 mediates more oxalate absorption by sulfate-oxalate exchange, leading to NADPH oxidase (Nox) change and intracellular ROS generation. Overproduced ROS could lead to NF*κ*B activation, which upregulates OPN expression. OPN plays a crucial role in the adhesion process of COM to cells. Excess COM adhesion induces cell apoptosis or necrosis, producing vesicles and crystal nucleation.

The present study results indicate that high expression of Slc26a6 in NRK cells could absorb more oxalate into cells and increase COM crystal adhesion (Figure 8(a)). The interaction between COM and the cell membrane leads to cell injury, resulting in more ROS production and NF*κ*B/OPN pathway activation in NRK cells, leading to more COM adhesion (Figure 8(b)) [28, 43]. The cellular studies indicate that low Slc26a6 expression levels in the kidney may protect it from urolithiasis formation.

Furthermore, we transfected lv-siRNA into rats' kidneys to identify SOD generation. The TUNEL assay showed NF*κ*B and OPN expression level changes and crystal formation. Because Slc26a6 is mainly expressed in both the duodenum and kidney, subcapsular injection was used to study the kidney separately [22]. After infection for 2 weeks, an IHC assay was performed, and the results indicate that the expression of Slc26a6 decreased significantly in the kidney but was not altered in the duodenum. Furthermore, the other results were consistent with the in vitro study.

There are some limitations to our study, which are worth mentioning. Firstly, in the present study, we just did an in vitro and vivo study, so further clinical verification is necessary. Secondly, more studies focusing on the association between oxalate transportation and other molecular proteins, which we did not investigate in this study, are needed [44]. Thirdly, we only downregulated the expression of Slc26a6, so further study knocking out the gene is needed. Fourthly, larger numbers of animal experiments are needed in future to verify the Slc26a6 function.

## 5. Conclusion

In conclusion, we propose that downregulation of Slc26a6 expression attenuated ROS production to reduce crystal formation.

## Data Availability

The data of the materials and methods and conclusions to support the findings of this study are included within the article. If any other data may be needed, please contact the corresponding author upon request.
